# Plasma neurological biomarkers as a measure of neurotoxicity in pediatric dental general anesthesia: a prospective observational feasibility study

**DOI:** 10.1007/s40368-024-00884-9

**Published:** 2024-04-22

**Authors:** S. Chakithandy, H. Nazzal, M. Matoug-Elwerfelli, S. Narasimhan, S. Uddin, K. S. Prabhu, L. Zarif, N. Mumtaz, A. Sharma, M. Al-Khelaifi

**Affiliations:** 1 Pediatric Anaesthesiology Department, Sidra Medicine, Doha, Qatar; 2 Hamad Dental Centre, Hamad Medical Corporation (HMC), Doha, Qatar; 3 College of Dental Medicine, QU Health, Qatar University, Doha, Qatar; 4 Translational Research Institute, Hamad Medical Corporation, Doha, Qatar; 5 Anaesthesiology Department, Weill Cornell Medicine, NewYork-Presbyterian Hospital, New York, USA; 6 Pediatric Anaesthesiology Department, Hamad Medical Corporation, Doha, Qatar

**Keywords:** Biomarkers, General anesthesia, Neurotoxicity, Pediatric dentistry

## Abstract

**Purpose:**

Neurotoxicity concerns have been raised over general anesthesia and sedation medication use in children. Such concerns are largely based on animal studies, historical anesthetic agents, and assessment tools, thus warranting further investigations. Blood biomarkers in detecting neuronal inflammation and apoptosis are novel methods for detecting neuronal damage. Therefore, the aim of this feasibility study was to assess the usefulness of the levels of four plasma biomarkers in dental general anesthesia (DGA) as surrogate markers of neurotoxicity in children. The secondary aim was to compare changes in motor manipulative skills pre- and post-anesthetic exposure.

**Methods:**

This single-center prospective observational study included 22 healthy children aged between 3 and 6 years old who underwent DGA. Subclinical neurotoxicity was measured with a panel of four plasma biomarkers: Caspase-3, neuron-specific enolase (NSE), neurofilament light chain, and S100B at three time points (1; at start, 2; end and 3; on recovery from DGA). The Skillings–Mack test was used to identify the difference in the biomarker levels at three time points. Motor manipulative score assessment, prior and two weeks after DGA was also performed.

**Results:**

A total of 22 study participants (mean age = 5 ± 1 years) were included with a median DGA duration of 106 ± 28 min. A reduction in Caspase-3 levels was recorded, with pairwise comparison over three time points, reporting a statistical significance between time point 2 vs. 1 and time point 3 vs. 1. Although fluctuations in NSE levels were recorded, no significant changes were found following pairwise comparison analysis. Among other biomarkers, no significant changes over the three periods were recorded. Furthermore, no significant changes in manipulative motor scores were reported.

**Conclusion:**

Caspase-3 reduced significantly in the short time frames during day-care DGA; this might be due to the relatively short anesthesia duration associated with dental treatment as compared with more extensive medical-related treatments. Therefore, further studies on Caspase-3 as a potential biomarker in pediatric DGA neurotoxicity are required to further ascertain results of this study.

## Introduction

The use of pharmacological behavior management, in the form of sedation and general anesthesia (GA), especially in managing young and uncooperative children requiring dental treatment, mainly as a result of dental caries, is widely used in pediatric dentistry. According to a recent UK report, more than 33,000 children under the age of nine underwent dental extractions in a hospital, of which 87% were due to tooth decay between March 2018 and March 2019 (Getting It Right First Time 2021).

Although based on animal studies, anesthetic and sedation medication safety concerns, largely related to neurotoxicity, have sparked significant attention (Jevtović-Todorović et al. 1998). Anaesthesia-induced developmental neurotxicity (AIDN), as a result of exposure to commonly used anesthetic and sedative agents during early childhood, is a widely discussed and debatable clinical issue with a significant public health-bearing (Levy et al. 2016; Vutskits and Davidson 2017). Adverse complications, such as neurotoxic deficits, neurocognitive impairments and behavioral abnormalities, have been reported (Levy et al. 2016). This ongoing clinical concern was initially sparked by the results of long-term behavioral changes in non-human primates following anesthesia exposure under experimental conditions (Jevtović-Todorović et al. 1998). Furthermore, a review of several animal studies concluded several neurological abnormalities, such as alterations in synaptic density and dendritic architecture, a reduction in trophic factors, neuronal and oligodendrocytic cell death, and long-term neurobehavioral abnormalities (Lin et al. 2014).

Despite ample experimental animal data (mainly on rodents) of sufficient clinical translation concluding that early-life exposure to GA may result in long-lasting neural and cognitive deficits, limited conclusive human studies are available on the question of anesthetic neurotoxicity partly because of a lack of an objective criteria for the suspected neurotoxicity. A recent multi-center randomized controlled clinical trial has proven that dental general anesthesia (DGA) of less than one h duration in children below 5 years of age did not alter the neurodevelopmental outcome in comparison to the awake-regional anesthesia (McCann et al. 2019). A plausible explanation is the short duration of the anesthetic.

Biomarkers have been used as surrogate tests of the neuronal outcome in detecting neuronal inflammation, neuroglail apoptosis, myelin and neuronal damage, following traumatic brain injury (Peacock et al. 2017). These biomarkers could be of clinical relevance as they are raised within a close time frame of the insult, thus giving a prognostic and possibly diagnostic prediction immediately after injury. The assessment of specific biomarkers as a neuronal test outcome following anesthesia exposure in young children is regarded as a novel path of inquiry. Indeed, establishing a reliable objective measure for identifying peri-operative subclinical neurological damage is of paramount importance in relation to pediatric anesthesia.

Within the literature, a high odds ratio for predicting post-operative cognitive dysfunction for specific established markers, such as neuron-specific enolase (NSE) and S100B protein measurements, have been reported (Silva et al. 2016; Stojanovic Stipic et al. 2017). Neurofilament light chain (NfL) has also been used to assess mild brain injury following trauma (Shahim et al. 2017) and anesthetic exposure (Deiner et al. 2020). Other prospective studies measuring serum Caspase-3 levels in the blood and cerebrospinal fluid related to anesthetic exposure also reported variable changes within the tested biomarkers following anesthesia exposure (Balasubramanian et al. 2021). Therefore, the primary aim of this study was to measure the usefulness of four plasma biomarkers (Caspase-3, NSE, NfL, and S100B protein) as a measure of subclinical neurotoxicity, in healthy children aged between 3 and 6 years following exposure to DGA. The secondary aim was to compare changes in motor manipulative skills pre- and post-anesthetic exposure.

## Materials and methods

### Study design and ethical approval

This study was a prospective, observational single-centre study conducted according to the principles expressed in the Declaration of Helsinki (World Medical Association 2013). Ethical approval was obtained from the Medical Research Centre and the Institutional Review Board of Hamad Medical Corporation, Qatar (MRC-01-19-160). Parents/legal guardians of children aged between 3 and 6 years old, with a medical history consistent with stages I and II of the American Society of Anesthesiologist (ASA), on Hamad Dental Hospital’s DGA waiting list were approached and consented to take part in this study. Informed consent was obtained from parents/legal guardians during pre-DGA assessment appointment.

### Inclusion and exclusion criteria

Healthy (ASA I and II) pediatric patients aged between 3 and 6 years old were included in this study. Excluded patients included; classified as ASA III and above, children having neurological or hepatorenal diseases or medications for the same, children outside the defined age range, or whom parents/legal guardian did not consent.

### Data collection

During the pre-DGA assessment appointment, patient’s demographic characteristics including age, sex, weight, previous anesthesia exposure, and duration of anesthesia were recorded. A pre-anesthetic motor manipulative score assessment was performed prior to the DGA. Three milliliter whole blood samples were drawn using 20 Gage Intravenous (IV) cannula under the aid of ultrasound guidance at three different time points as follows: Time point 1 (start) immediately post-anesthesia induction and IV cannulae insertion. Time point 2 (end) at the end of the surgery before extubation via the same IV cannula. Time point 3 (recovery) on post-operative recovery before discharge via the same IV cannula.

All children received inhalation induction followed by deepening of anesthetic with Propofol and Rocuronium, and maintenance with Sevoflurane. Opioids and other analgesics were added at the discretion of the individual anesthetist. The blood samples were collected in individual collection tubes (Vacutainer, Becton Dickenson; Plymouth, UK), de-identified, processed, and stored at −80 °C. The samples were then shipped on dry ice to the ImmunoArray laboratory (Translational Research Institute, Hamad Medical Corporation, Qatar) for testing. The obtained blood samples were analyzed for relevant biomarkers with target proteins detected using enzyme-linked immunosorbent assay (ELISA) based technology.

### Plasma biomarkers

Plasma levels of NSE, NfL, S100B, and Caspase-3 were tested using ELISA and detected using Spark^®^ multi-mode microplate reader (TECAN, Switzerland). Based on the relevant target proteins, specific kits including Human Enolase 2 quantitative ELISA kit (R&D Systems, USA), Human NfL (LSBio, Seattle), S100B ELISA kit (EMD Millipore, Missouri) and Caspase-3 ELISA kit (RayBiotech, USA) were used according to manufacture instructions. All plasma samples were tested in duplicates with the average concentrations obtained via a four parameter logistic (4PL) regression curve equation from the standard curve. The acceptance criteria for NSE included replicate samples varying less than 10% (coefficient of variation, CV), recovery of 85–111% and a regression curve linearity range of 92–101%. For S100B, the acceptance criteria included replicate samples varying less than 15% (CV) and a recovery of 94%. The acceptance criteria for NfL included replicate samples varying less than 10% (CV). The average concentrations for Caspase-3 were obtained via a log–log curve. The acceptance criteria included replicate samples varying less than 10% (CV), with a recovery of 95–146% and regression curve linearity range of 129–140%. The lower limits of detection of the tested serum proteins were as follows: NSE (0.02 ng/ml), NfL (6.2 pg/ml), S100B (2.7 pg/ml), and Caspase-3 (40 pg/ml).

### Motor manipulative score assessment

The motor manipulative score, fine motor sub-domains of significance in the ‘schedule of growing skills-2’ was performed in the dental clinic before and two weeks after DGA.

### Statistical analysis

The data were tabulated and analyzed statistically using IBM SPSS Statistics for Windows, version 26 (IBM Corp., Armonk, N.Y., USA). Simple descriptive statistics was used to represent the nature of the data. Shapiro–Wilk test revealed non-normal distribution of the data. Therefore, Skillings–Mack test (a generalized version of Friedman test) and Dunn post hoc test were used to identify the significant difference in the biomarkers levels at the three time points. Wilcoxon signed-rank test was used to find the difference in the pre- and two weeks post-fine motor assessment scores. A *p* value less than 0.05 was considered as significant.

## Results

Twenty-three children were recruited in this study between July 2020 and March 2021. Following exclusion of one patient (due to inability to obtain blood samples at any of the three time points), a total of 22 participants remained in the study (10 females and 12 males). Detailed descriptive statistics of patient’s demographics and study parameters, such as age, weight of the child and duration of the anesthesia, is presented in Table 1. The median age was 5 years [IQR for the difference in median (4–6)], the median weight was 18 kg [IQR for the difference in median (16.3–21.5)] and median anesthesia duration was 108 min [IQR for the difference in median (83–115)]. The median time for the collection of blood samples for time point 3 (recovery) was 35 min. Among the three time points, 64 samples were analyzed for the panel of four serum biomarkers. In two patients, recovery samples was not obtained. Thus, a total of 256 biomarker levels were analyzed.

**Table 1:** Descriptive statistics representing participants’ age, weight, and anesthetic duration

Participants parameters	Mean (SD)	Median	Percentile 25	Percentile 75
Age (years)	5 (1)	5	4	6
Weight (kg)	18.55 (3.47)	18.00	16.30	21.50
Anesthesia duration (min)	106 (28)	108	83	115

*Kg* kilograms, *min* minutes, *SD* standard deviation

### Plasma biomarkers

#### Caspase-3

**Fig. 1:**
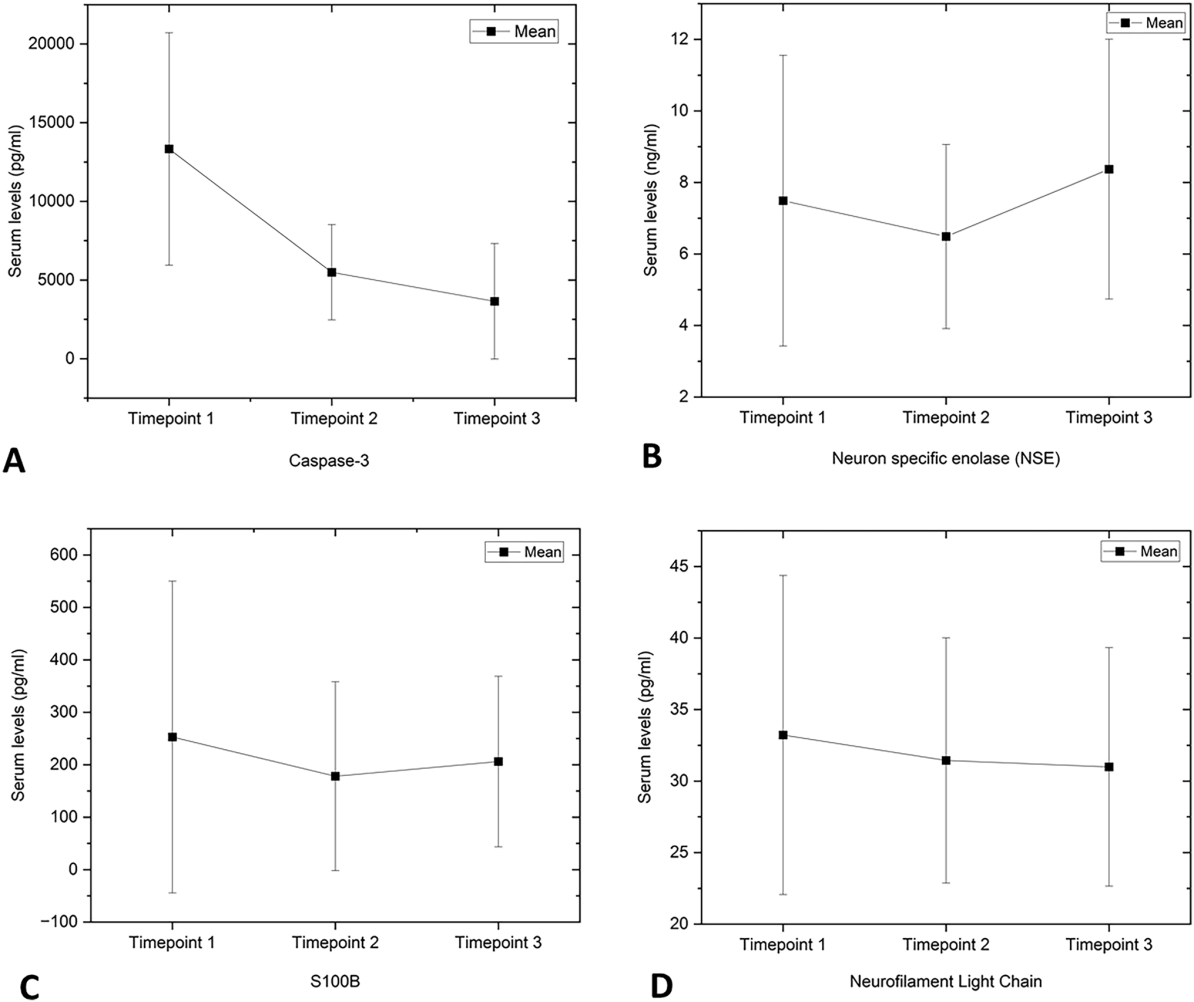
Line graphs representing mean (standard deviation) of tested serum levels biomarkers; **A** Caspase-3, **B** neuron-specific enolase, **C** S100B, and **D** neurofilament light chain at the three time points; (1) immediately post-anesthesia induction, (2) at the end of the surgery, and (3) post-operatively prior to discharge

A statistical significant reduction in Caspase-3 levels from time point 1 (start) to time point 2 (end) and time point 3 (recovery) was noted (Skillings–Mack test; 25.409, *p* < 0.001) as presented in Table 2 and Fig. 1A. Further pairwise comparison over the three time points, reported a significant difference between time point 2 vs. 1 and time point 3 vs. 1 (*p* < 0.001), as presented in Table 3.

**Table 2:** Statistical analysis of the tested plasma biomarker measurements across the three time points

Plasma biomarker (unit)	Time points	Mean (SD)	Median (IQR)	*p* value (Skillings–Mack test)
Caspase-3 (pg/ml)	1	13,331.50 (7559.36)	11,615 (8628, 14,848)^a^	*p* < 0.001*
	2	5493.05 (3099.16)	4927 (3002, 8393)^b^	
	3	3649.49 (3766.59)	2029 (594, 5878)^b^	
Neuron-specific enolase (ng/ml)	1	7.49 (4.16)	6.86 (5.78, 8.60)^a^	*p* = 0.013*
	2	6.49 (2.64)	5.93 (4.79, 6.86)^b^	
	3	8.37 (3.73)	6.8 (5.8, 10.3)^a^	
S100B (pg/ml)	1	253.01 (304.30)	125 (87, 242)	*p* = 0.26
	2	178.24 (184.30)	130 (92, 178)	
	3	206.16 (166.82)	156 (101, 262)	
Neurofilament light chain (pg/ml)	1	33.22 (11.41)	30 (27, 37)	*p* = 0.952
	2	31.44 (8.77)	30 (25, 35)	
	3	30.99 (8.56)	30 (26, 38)	

Different alphabetical superscripts denote statistically significant difference in the biomarker level measured at various time points. (1) Start: immediately post-anesthesia induction and IV Canula insertion, (2) end: at the end of the surgery before extubation, (3) recovery: post-operatively prior to discharge

*SD* standard deviation, *IQR* interquartile range, *statistically significant

**Table 3:** Pairwise comparison over the three time points for neuron-specific enolase and Caspase-3 using Dunn’s test (primary test used: Skillings–Mack test)

Biomarker	Pairwise comparison	Mean difference	*p* value
Neuron-specific enolase	Time 2 vs. Time 1	−1.00	0.350
	Time 3 vs. Time 1	0.88	0.518
	Time 3 vs. Time 2	1.88	0.090*
Caspase-3	Time 2 vs. Time 1	−7,838.45	<0.001
	Time 3 vs. Time 1	−9,682.01	<0.001
	Time 3 vs. Time 2	−1,843.55	0.186

#### Neuron-specific enolase (NSE)

Although a reduction in NSE levels from time point 1 to time point 2 (end), followed by a rise in levels to time point 3 (recovery) was recorded (Skillings–Mack test; 8.795, *p* = 0.0129) as presented in Table 2 and Fig. 1B, further pairwise comparison over the three time points found no significant difference between time point 3 vs. 2 (*p* = 0.090), as presented in Table 3.

#### S100B

Changes in mean plasma S100B levels between the three time points were noted whereby the mean baseline levels (start) decreased at time point 2 (end) and then rose again at time point 3 (recovery). However, no significant difference was found across the three time points (Skillings–Mack test; 2.706, *p* = 0.2585) as presented in Table 2 and Fig. 1C.

#### Plasma neurofilament light chain (NfL)

Although a clinically meaningful change in mean NfL levels between the three time points was reported, no significant difference was found across the three time points (Skillings–Mack test, 0.098, *p* = 0.9522). The mean baseline NfL levels (start) decreased at both time point 2 (end) and time point 3 (recovery) as presented in Table 2 and Fig. 1D.

#### Motor manipulative skill sub-domain SGS-2 score

Results of the manipulative motor score assessment (schedule of growing skills-2) revealed all scores within the expected age range, with exception of one patient. The latter reported a lower than expected motor manipulative scoring suggesting the potential of an unrecognized developmental delay. Overall, there was no significant difference between the pre-DGA (26.09 ± 5.21) and the post-DGA (26.25 ± 4.60) motor manipulative scores (Table 4).

**Table 4:** Differences in the patient’s pre- and post-DGA motor manipulative scores assessed using Wilcoxon signed-rank test

Fine motor assessment	Mean (SD)	Median	Percentile 25	Percentile 75	*p* value (Wilcoxon value)
Pre-anesthesia	26.09 (5.21)	28.00	27.00	28.00	0.952 (22)
Post-anesthesia	26.25 (4.60)	27.00	27.00	28.00	

*SD* standard deviation

## Discussion

Adverse long-term impact of anesthesia and sedative agents on the developing brain in young children, such as anesthesia-induced developmental neurotoxicity, is a widely discussed topic since the 2016 Food and Drug Administration (FDA) drug safety communication (FDA 2016). The results of a multi-center randomized controlled clinical study, comparing GA and awake-regional anesthesia (GAS trial) in infants aged 2 years, showed absence of neurotoxicity following GA of less than one h at 2- and 5-years follow-up (Davidson et al. 2016; McCann et al. 2019). The above study was in-line with the Paediatric Anaesthesia Neurodevelopment Assessment (PANDA) study, which assessed the developmental outcomes in matched-siblings (aged 8–15 years) with or without GA exposure, before the age of 3 years (Sun et al. 2016). Furthermore, the results of the Mayo Anaesthesia Safety in Kids (MASK) assessing neuropsychological of children aged either 8 to 12 or 15 to 20 years old with a history of no exposure, single exposure, or multiple GA exposure prior to the age 3 years reported no difference in intelligence quotient (IQ) scores between all groups. They further concluded that multiple, but not single, GA exposure could be associated with behavioral and learning difficulties (Warner et al. 2018). Therefore, neurotoxicity and cognitive changes associated with anesthetic exposure remains debatable (Vutskits et al. 2012).

The utilization of biomarkers as an outcome measure of anesthetic agents’ effect on neural tissues has been reviewed to assist in the development in safe and effective anesthetic strategies for young children (Levy et al. 2016). Overall, serum biomarkers have been shown to be a reliable indicator of a biological processes but not necessarily a clinical endpoint (Strimbu and Tavel 2010). Serum biomarkers validity as a predictive tool for neurological damage in mild traumatic brain injury cases has been proven (Shahim et al. 2016; Lee et al. 2021). At a cellular level, they are regarded as surrogate markers for physiological processes, such as apoptosis, glial/astrocyte damage, neuronal damage, neural inflammation, and synaptogenesis inhibition. The baseline values of these biomarkers vary with genetic and environmental factors. Despite variable baseline biomarker levels, an increase or decrease in these surrogate markers with anesthesia and surgery denotes protein activity changes within the neurons. Each biomarker must be correlated in conjugation with a range of other biomarkers, hence a panel of biomarkers are recommended.

According to the literature, changes in serum Caspase-3 and NfL levels have been reported following GA exposure (Balasubramanian et al. 2021). Additionally, specific biomarkers have received considerable interest, such as NSE and S100B protein, for predicting post-operative cognitive dysfunction (Silva et al. 2016; Stojanovic Stipic et al. 2017). Therefore, changes in serum levels of four plasma biomarkers: Caspase-3, NSE, NfL and S100B protein were assessed in this study as surrogate markers of neurotoxicity in children. Overall results of this study report a reduction in Caspase-3 levels in otherwise healthy children aged between 3 and 6 years old following short duration DGA exposure, thus suggesting an absence of subclinical neurotoxicity.

Serum Caspase-3 protein, also known as CPP32 or apopain, is a member of the endoproteases (cysteine-aspartic proteases) family (Asadi et al. 2021). Caspase-3 has been identified as a key biomarker of apoptosis, activated in apoptotic cells via both extrinsic and intrinsic pathways. Furthermore, non-apoptotic functions, such as neural development, tissue differentiation, and regeneration, have been correlated to Caspase-3 levels (Shalini et al. 2015; Asadi et al. 2021). Caspase-3 levels denote the rate of apoptosis with cell death caused by proteolysis of many cellular proteins (Yang et al. 2020). Of clinical importance is the reported rapid increase in Caspase-3 activity post insult, which has been linked to stroke and traumatic brain injury in the adult population (Lorente et al. 2015; Glushakova et al. 2017). Raised serum Caspase-3 levels have also been seen in degenerative neurological conditions. Therefore, levels of Caspase-3 are measured as a marker of prognosis in intensive care units (Balasubramanian et al. 2021).

In this study, Caspase-3 persistently reduced after anesthetic exposure suggesting no neurological damage within the anesthetic dosages used in this study, in this age group following short anesthetic exposures (106 ± 28 min). These findings correlate with the primate study, in which exposure to isoflurane induced a significant increase in neurodegenerative apoptotic biomarker S100B and Caspase-3 levels as opposed to the equipotent exposure of sevoflurane (Liang et al. 2010). The immediate cause for dynamic changes in serum Caspase-3 noted in our study is hard to pinpoint without associated nucleotide imaging of the brain. However, findings of this study reiterate the usefulness of this biomarker as a neuromonitoring tool for the various anesthetic agents and various durations of anesthesia in this age group.

Serum NSE biomarker, a critical glycolytic protein, is considered as a well-known biomarker of neuronal damage (Shaik et al. 2019). The glycolytic protein pathway of NSE is known to increase following brain injury, trauma, and epilepsy (Shaik et al. 2019; Lee et al. 2021). Normal serum levels of NSE are within the range of 2–20 ng/ml, with a pathological level recorded at more than 30 ng/ml (Cata et al. 2011). Results of this study revealed that NSE levels were within normal serum levels, with a lower detection limit of 6.49 to 8.37 ng/ml. Therefore, no clinical significance can be attributed to NSE changes since the overall levels were within the normal physiological range reported previously.

S100B protein, a calcium-binding protein, is known to be present in astrocytes and glial cells, hence a potential role as a biomarker in cases of degenerative and inflammatory neurological diseases (Cata et al. 2011). Additionally, Serum NfL, a neuron-specific marker of neurological injury is reported to be the most abundant and soluble proteins of the neurofilament family (Thebault et al. 2020). This feasibility study reported no statistically significant changes in S100B and NfL levels among the three measured time points. Such results could be attributed to the short post-operative time at which the blood samples were collected. Although longer post-operative sample collection might show different results, obtaining blood samples post-DGA might be difficult in such young group of patients.

Of clinical interest, child recovery following DGA could be associated with variable interchangeable behavioral disturbances, such as post-anesthetic agitation, delirium, and excitement (Vlajkovic and Sindjelic 2007; Reduque and Verghese 2012). As this study included children aged between 3 and 6 years old, only fine motor sub-domains of significance in the ‘schedule of growing skills-2’ were used to assess neurological changes effecting fine motor function. All included children were scored twice; just before and then two weeks after DGA. The maximum score possible was 28 (21–23 expected for 3-year-old, 24–26 expected for 4-year-old and 27–28 expected for 5 years old). All patients, except one, scored within the expected ranges indicating no effect on children’s motor function two weeks post-DGA.

Limitations of this study include the lack of a control group, short assessment period and low sample size in which the effect of confounding factors, such as type of dental treatment, pre-existing pain, stress, and post-operative time, were not assessed and could have affected the outcomes of this study. Although patients with previous experience of GA were not excluded from this feasibility study, only one patient had such experience. Future controlled studies should be conducted to further understand the effect of multiple anesthetic exposure. Additionally, lack of long-term sampling of the biomarkers (i.e., sample collection at regular intervals, such as 3, 6, 12, and 24 h) might provide better assessment to the changes in serum level of biomarkers. Despite the above, this study adds to the scarce human studies on the safety neurotoxicity of DGA agents, especially in the dental pediatric field where the majority of procedures do not exceed 1–2 h. This study as such can be considered as a stepping stone to a larger study looking at biomarkers for neurotoxicity to anesthesia exposure of three h or more in duration. Therefore, future prospective well-designed studies of sufficient sample size are required to ascertain which serum biomarkers can be used to compare toxic effects of GA agents in comparison to patients receiving dental treatment under local analgesia. Furthermore, studies assessing the effect of GA on children below the age of 3 years are of interest as neurotoxicity could be higher due to immaturity of neural tissue. These future studies assessing serum biomarkers should consider including NSE and Caspase-3 as both have been shown to be sensitive to neurological insult in the immediate pre-operative period in our this study. Additionally, the primary end point to look for is also important and more objective end points, such as biomarkers and neuroimaging, is recommended in future studies.

## Conclusion

Within the limitations of this study design, the overall results indicate that serum Caspase-3 changes dynamically in the short peri-operative period of day care dental surgery in a sample of healthy children aged between 3 and 6 years old. The significant reduction in Caspase-3 levels suggests an absence of neurotoxicity with short sevoflurane-based anesthesia in this patient group over a maximum exposure of 2 h.

## Data Availability

The data that support the findings of this study are available from the corresponding author, upon reasonable request.
